# Simultaneous Bilateral Lesser Tuberosity Fracture Fixation Using Suture Osteosynthesis in Porotic Bone: A Case Report

**DOI:** 10.7759/cureus.105982

**Published:** 2026-03-27

**Authors:** Nazim Sifi, Khalid Benokba, Nicolas Robial

**Affiliations:** 1 Orthopaedic and Trauma Surgery Unit, Hôpital Pasteur, Colmar, FRA

**Keywords:** biceps tenodesis, bilateral, lesser tuberosity fracture, osteoporosis, suture fixation

## Abstract

Isolated bilateral fractures of the lesser tuberosity of the humerus represent an exceptionally rare subset of proximal humerus fractures. A 66‑year‑old active woman with chronic steroid‑induced osteoporosis presented with bilateral shoulder pain after a ground‑level fall down a step. Clinical examination demonstrated marked bilateral loss of internal rotation strength with positive belly‑press and bear‑hug tests, whereas external rotation was preserved. Standard radiographs, CT, and MRI confirmed displaced bilateral lesser tuberosity avulsion fractures in porotic bone, with the subscapularis tendon attached to the fragments and an otherwise intact rotator cuff. Given the degree of displacement, functional impairment, and high functional demands, a single‑stage bilateral open surgical procedure was performed in the beach‑chair position through a deltopectoral approach, consisting of long head biceps tenotomy with tenodesis using a 5‑mm titanium anchor (ArthroVims®; VIMS, Villeneuve-lès-Bouloc, France) and suture‑based fixation of the lesser tuberosity fragments using nonabsorbable Smartloop® and SmartTape™ sutures (FX Solutions, Viriat, France), tailored to the small, porotic fragments. Postoperative management consisted of sling immobilization for six weeks, immediate pendulum exercises, passive external rotation limited to 0° from week 3, and active mobilization from week 6, resulting in excellent shoulder function at only three months’ follow‑up. This case illustrates the feasibility of simultaneous bilateral surgery in an osteoporotic patient, with fixation adapted to poor bone quality (suture fixation rather than screw fixation) and systematic management of the long head of the biceps in the setting of pulley involvement. Only three cases of isolated bilateral lesser tuberosity fractures have been reported to date, underscoring the rarity of this injury pattern, the value of advanced imaging, and the role of operative treatment in displaced fractures.

## Introduction

Isolated fractures of the lesser tuberosity are rare injuries within the spectrum of proximal humerus fractures, with an estimated annual incidence of approximately 0.46 per 100,000 [1], and are most often described in case reports or small case series. In most reported cases, the lesser tuberosity is involved unilaterally or in the setting of asymmetric bilateral injuries, such as bilateral fracture‑dislocations with a lesser tuberosity fracture on one side and a greater tuberosity fracture on the other [2], rather than truly isolated bilateral lesser tuberosity fractures. Isolated bilateral lesser tuberosity fractures are even more exceptional, with only a few similar cases reported, including a young athlete after low‑energy trauma [3], a young adult with seizure‑related injury [4], and an adult with alcohol withdrawal seizures [5]. Across these reports, the common pathomechanism is an avulsion of the lesser tuberosity by a sudden forceful contraction of the subscapularis during abduction and external rotation of the shoulder. In the present case, a ground‑level fall with likely forced external rotation in an osteoporotic patient on long‑term corticosteroids fits this biomechanical model, further aggravated by compromised bone quality.

## Case presentation

A 66‑year‑old right‑hand‑dominant retired woman presented with bilateral shoulder pain after a ground‑level fall down a step. She was physically active with moderate sports participation and had a medical history of hypertension, non‑insulin‑dependent diabetes mellitus, and chronic adrenal insufficiency treated with long‑term corticosteroids. Two weeks after the injury, she was referred for persistent bilateral shoulder pain and functional loss. On clinical examination, active and passive external rotation of both shoulders were preserved, whereas there was marked bilateral weakness in internal rotation with positive belly‑press and bear‑hug tests. There was no obvious deformity and no clinical evidence of neurological or vascular compromise, and no associated injuries were identified.

**Figure 1:**
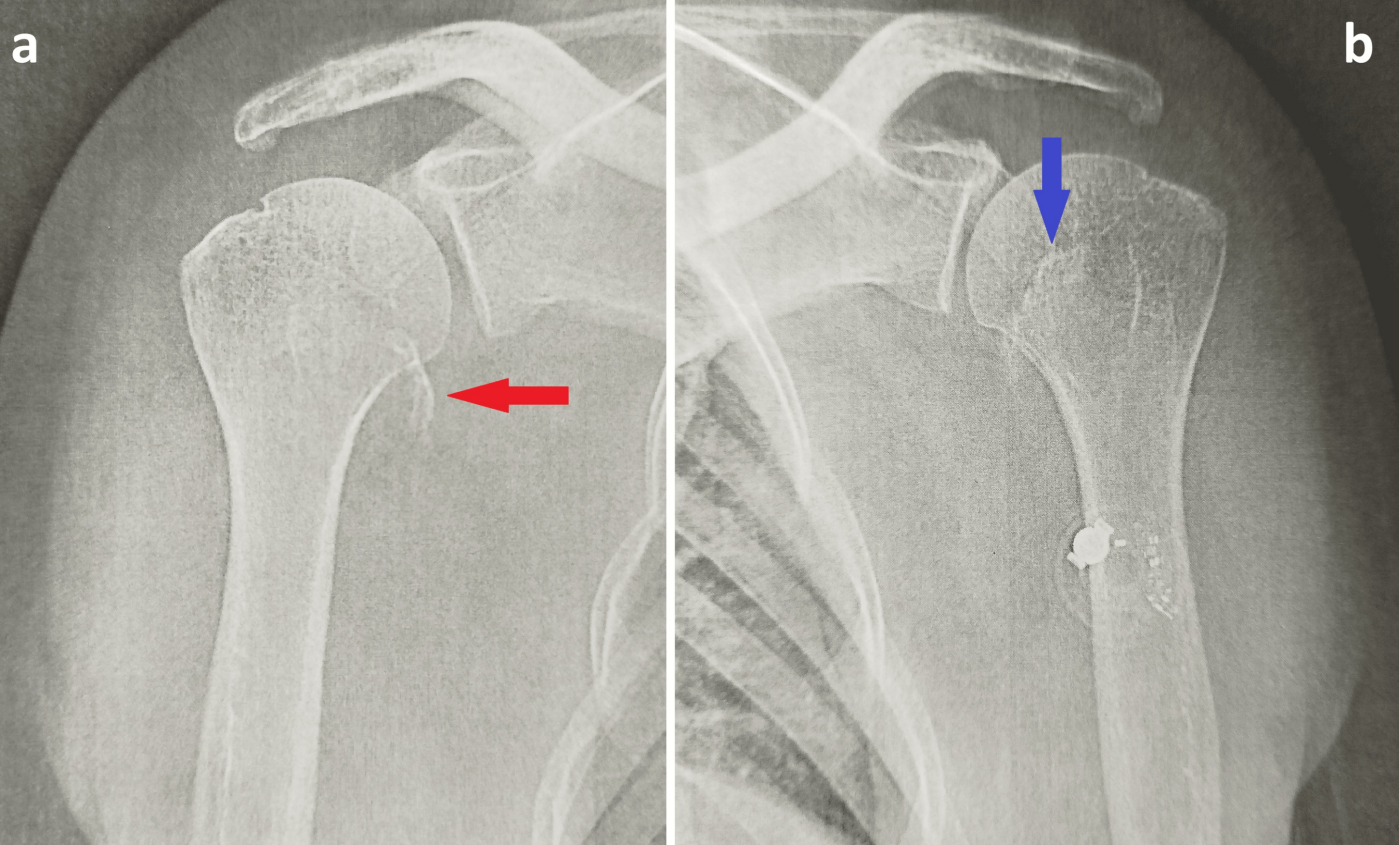
Anteroposterior radiographs of the right (a) and left (b) shoulders demonstrating displaced lesser tuberosity avulsion fractures (red and blue arrows), which are particularly subtle on the left side (blue arrow).

Standard anteroposterior radiographs demonstrated bilateral abnormalities involving the lesser tuberosities but did not clearly delineate the extent of the injury (Figure 1).

**Figure 2:**
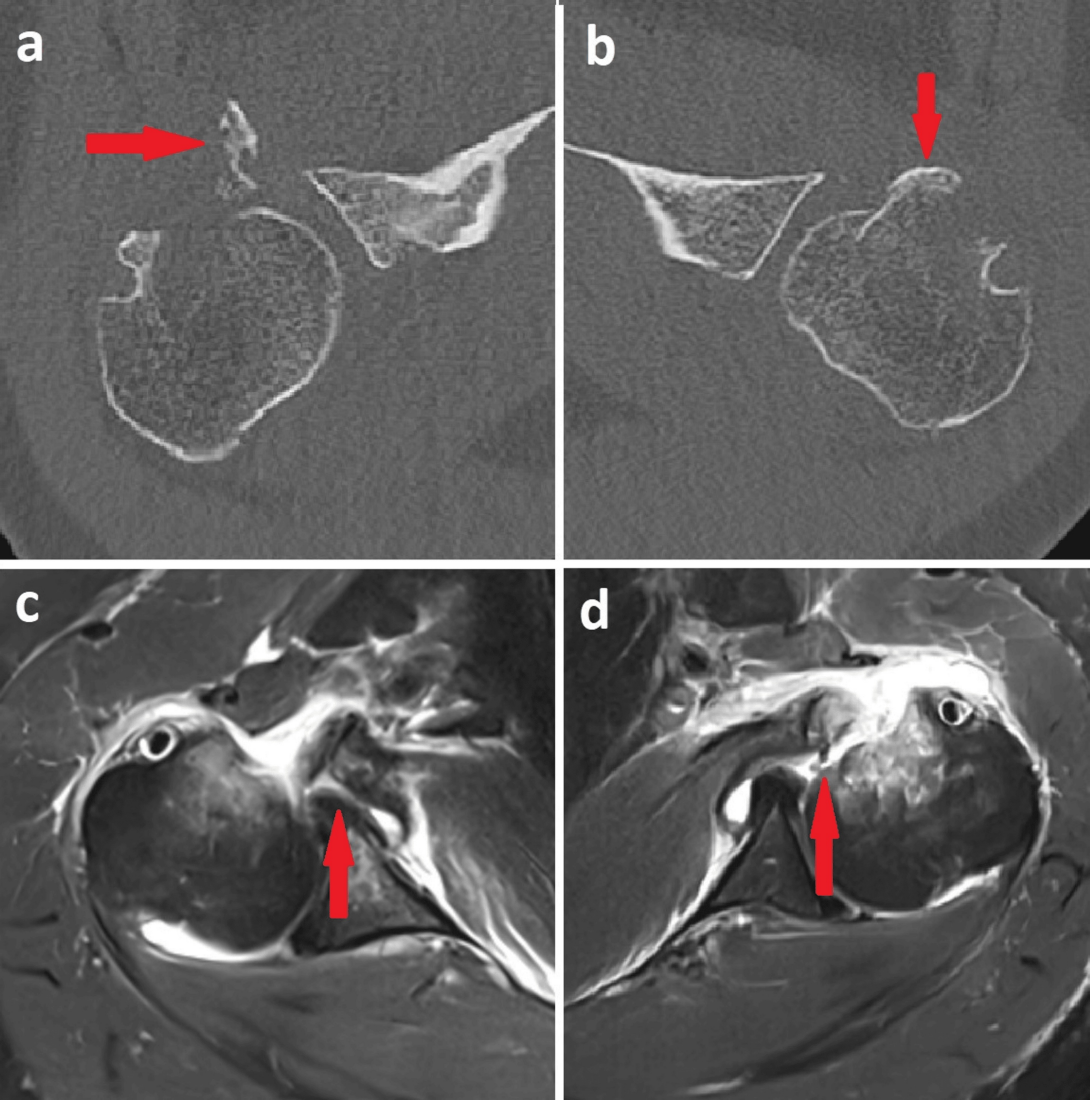
CT (a,b) and MRI (c,d) of the right and left shoulders showing displaced lesser tuberosity fragments (>5 mm) with the subscapularis tendon attached (red arrows) and diffuse osteopenic bone.

Complementary CT and MRI of both shoulders confirmed bilateral displaced lesser tuberosity avulsion fractures with more than 5 mm of fragment displacement, with the subscapularis tendon attached to the bony fragments and an otherwise intact rotator cuff (Figure 2).

Bone quality appeared markedly reduced, consistent with corticosteroid‑induced osteoporosis. Treatment options were discussed with the patient, taking into account her age, degree of displacement, bone quality, and high functional expectations. In view of the significant displacement (>5 mm), poor bone stock, and the need to restore bilateral shoulder function, operative treatment of both shoulders was selected. ​

**Figure 3:**
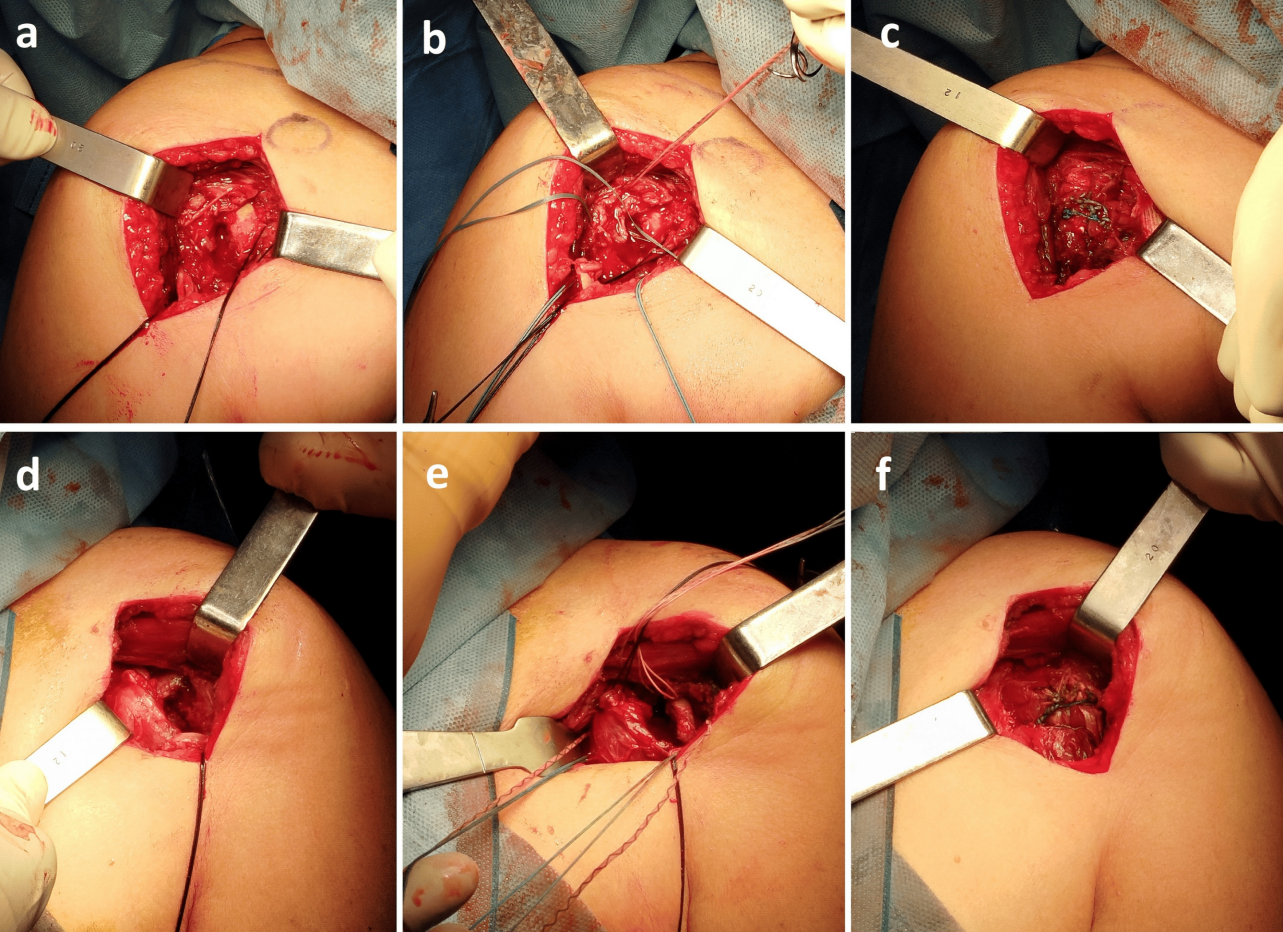
Intraoperative photographs of the right (a,b,c) and left (d,e,f) shoulders demonstrating suture based fixation of the lesser tuberosity fragments and tenodesis of the long head of the biceps tendon.

The patient was positioned in the beach‑chair position, which allowed sequential access to both shoulders during the same anesthetic event. After skin preparation and draping of the right shoulder, a standard deltopectoral approach was used to obtain anterior exposure of the proximal humerus. The long head of the biceps tendon was identified and isolated; a tenotomy was performed, followed by tenodesis using a 5‑mm titanium anchor (ArthroVims®; VIMS, Villeneuve-lès-Bouloc, France). Intraoperatively, the bone was clearly porotic, and the lesser tuberosity fragment was too small and thin to allow stable screw fixation. Therefore, fixation of the lesser tuberosity was achieved with nonabsorbable suture osteosynthesis using Smartloop® and SmartTape™ sutures (FX Solutions, Viriat, France), passed through the subscapularis tendon and fragment to obtain secure reduction and compression without risking fragment comminution. Intraoperative testing confirmed satisfactory stability of the construct. The same sequence, deltopectoral approach, long head biceps tenotomy‑tenodesis, and suture‑based fixation of the lesser tuberosity, was then replicated on the left shoulder during the same anesthetic session (Figure 3).

**Figure 4:**
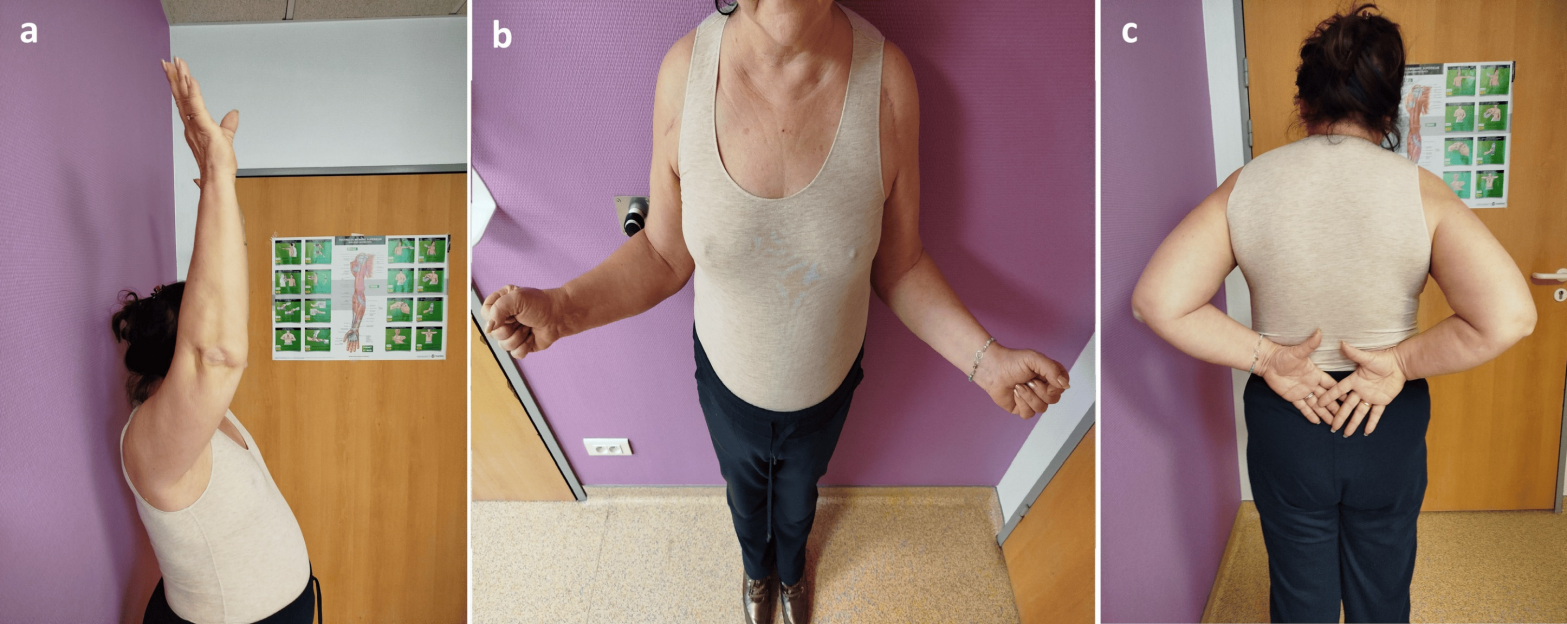
Clinical photographs at the three-month follow-up showing active forward elevation (a), external rotation (b), and internal rotation (c), illustrating the excellent functional recovery.

At the end of the procedure, the patient was placed in bilateral shoulder immobilizers (slings) for six weeks to protect the repair during early healing. Pendulum exercises were initiated on the first postoperative day and carefully demonstrated to the patient. Passive external rotation, strictly limited to 0°, was started at three weeks to protect the subscapularis repair and fixation while maintaining joint mobility. Active shoulder mobilization was initiated at six weeks, once satisfactory radiographic and clinical stability of the repairs was obtained, allowing progressive functional recovery without risk of secondary displacement. At the three-month follow‑up, bilateral shoulder function was already highly satisfactory, with forward elevation (FE) of 160-170°, external rotation (ER1) of 45-55° with the arm at the side, and internal rotation (IR) to T9-T10 (Figure 4).

## Discussion

Displaced fractures of the lesser tuberosity result in detensioning of the subscapularis tendon, leading to pain, weakness in internal rotation, and a risk of posterior shoulder instability or subcoracoid impingement. Previous reports have emphasized that diagnosis is frequently missed on standard radiographs, particularly in the absence of a true axillary view, and that CT or MRI is often required to identify fragment displacement and subscapularis involvement [3,5]. In the present case, the combination of radiographs, CT, and MRI was crucial to confirm the bilateral nature of the injury, quantify displacement, assess bone quality, and verify the integrity of the remaining rotator cuff, all of which guided the treatment strategy. Regarding treatment, fragment displacement greater than 5 mm or angulation greater than 45°, combined with functional deficit in internal rotation, is widely considered a primary indication for operative fixation, often outweighing chronological age alone [4].

In the literature, Zuchelli et al. reported operative fixation of only the displaced side (double‑row suture anchor construct plus screw fixation) with nonoperative treatment of the contralateral nondisplaced fracture, achieving symmetric function at one year [4], whereas Nowell et al. performed bilateral open reduction and internal fixation using a double‑row construct in a highly active adolescent, with full return to sports [3]. Conversely, Flaherty et al. described successful nonoperative management of minimally displaced bilateral lesser tuberosity avulsion fractures related to alcohol withdrawal seizures [5]. In the present case, the decision to operate on both shoulders rested on three key factors: significant displacement of both fragments, bilateral functional deficit in internal rotation, and severe steroid‑induced osteopenia, where secondary displacement or nonunion would have been particularly detrimental to the patient’s autonomy. The fixation technique must be tailored to fragment morphology and bone quality. When the fragment is large and bone stock is good, direct screw fixation can be considered [6], whereas in the presence of small or thin fragments or osteoporotic bone, suture‑based fixation and/or suture anchors are recommended to avoid fragmentation and loss of fixation. In this patient, nonabsorbable suture osteosynthesis of the lesser tuberosity was preferred, providing robust fixation while avoiding the risk of comminution or collapse associated with screw placement in markedly osteoporotic bone. This approach restores the subscapularis footprint, reduces the risk of nonunion or fixation failure, and optimizes shoulder biomechanics, particularly internal rotation strength and prevention of posterior instability or subcoracoid impingement.

The superior portion of the lesser tuberosity contributes to the biceps pulley complex and the stability of the long head of the biceps tendon, and fracture‑avulsions involving this region are typically associated with biceps tendon instability or dislocation. In this context, several authors advocate tenotomy or tenodesis of the long head of the biceps as a more reliable option than simple reduction of the tendon into its groove, particularly in older or less cosmetically concerned patients [7]. In the current case, systematic long head biceps tenotomy‑tenodesis on both shoulders was chosen to address the pulley involvement and provide durable pain relief and stability in an elderly, functionally demanding patient for whom cosmetic appearance was of secondary importance.

Another distinctive aspect of this case is the choice of single‑stage bilateral surgery under one anesthetic. Nowell et al. reported a similar approach in a skeletally immature, high‑demand patient with healthy bone [3], whereas Zuchelli et al. treated only one side operatively and the contralateral side nonoperatively [4]. The present report demonstrates that a single‑stage bilateral approach is also feasible in an osteoporotic patient, provided meticulous preoperative planning, controlled operative time, and a clearly defined rehabilitation protocol. The beach‑chair position and sequential bilateral deltopectoral approaches allowed satisfactory visualization and fixation of both lesions during the same procedure.

Postoperative rehabilitation plays a decisive role in functional outcome. Protocols described by Zuchelli and Nowell generally recommend four to six weeks of immobilization, early pendulum exercises, and gradual progression to active motion and strengthening, with near‑complete recovery over 6-12 months [3,4]. In this case, six weeks of sling immobilization provided protection of the repair, while immediate pendulum exercises helped to limit stiffness. Passive external rotation, strictly controlled to 0° from week 3, followed by active mobilization from week 6, offered a balanced compromise between protection of the suture fixation and prevention of stiffness, allowing very satisfactory functional recovery as early as three months postoperatively (FE: 160-170°, ER1: 45-55°, IR: T9-T10). Notably, Flaherty’s report did not provide a detailed rehabilitation protocol [5], emphasizing the value of documenting this phase, especially in bilateral injuries in patients at risk for osteoporosis.

## Conclusions

This case highlights that in osteoporotic patients with displaced bilateral lesser tuberosity fractures, single‑stage bilateral operative management combining suture‑based fixation of the lesser tuberosity, systematic long head biceps tenotomy‑tenodesis, and structured rehabilitation constitutes a treatment option consistent with current literature and capable of providing excellent functional outcomes.
